# Factors Associated with Unmet Need for Family Planning among Married Reproductive Age Women in Toke Kutaye District, Oromia, Ethiopia

**DOI:** 10.1155/2021/5514498

**Published:** 2021-03-26

**Authors:** Abebe Tadesse G/Meskel, Habtamu Oljira Desta, Elias Teferi Bala

**Affiliations:** ^1^West Shoa Zonal Health Department, Oromia Region, Ambo, Ethiopia; ^2^Department of Public Health, College of Medicine and Health Sciences, Ambo University, Ambo, Ethiopia

## Abstract

**Background:**

It is estimated that more than 142 million married women in developing countries have an unmet need for family planning. This study is aimed at identifying factors associated with the unmet need of family planning among married women of reproductive age in Toke Kutaye district, Ethiopia in 2019.

**Methods:**

A community-based cross-sectional study was conducted in Toke Kutaye district from March 1–30, 2019. A systematic random sampling technique was used to select 494 reproductive-age women who were married during data collection. Data were collected using a pretested structured questionnaire. Bivariate and multivariate logistic regression analyses were used to assess factors associated with the unmet need of family planning at 95% CI with a *p* value of ≤ 0.05.

**Result:**

The prevalence of unmet need for family planning in the Toke Kutaye district was 23.1% [95% CI (19.2-26.7)], with 15.2% for spacing and 7.9% for limiting. Women's education [AOR, 3.64, 95% CI: 1.43-9.25], number of living children [AOR, 2.63, 95% CI: 1.37-5.05], husband disapproval of family planning [AOR, 3.68, 95% CI: 2.20-6.16], and discussion with healthcare providers on family planning [AOR, 0.20, 95% CI: 0.13-0.37] were significantly associated with unmet need for family planning.

**Conclusion:**

The prevalence of unmet need for family planning was high. Therefore, program managers, partners, and health workers should work to address the gaps in maternal education, the number of living children, partner disapproval of family planning, and discussion on family planning issues through enhancing female education, awareness on family planning, and male involvement in family planning services.

## 1. Introduction

Unmet need for family planning is a statistical measure that calculates sexually active women who are not currently using any method of contraception and want to stop or delay their next birth by at least two years [1–5]. The sum of the unmet need for spacing and unmet need for limiting forms the total unmet need family planning [1, 6].

It is one of several frequently used indicators for monitoring family planning programs. Data on unmet needs can also help family planning programs target activities by identifying women who are at greatest risk of unintended pregnancy and more likely to accept a method than other nonusers [6, 7]. The sum of the unmet need for spacing and unmet need for limiting forms the total unmet need family planning [1, 6].

United Nations (UN) trend report of contraceptive use showed that worldwide in 2015, 12% of married women are estimated to have had an unmet need for family planning. This shows at least one in ten married women in most regions of the world has an unmet need for family planning. The level was much higher, 22%, in the least developed countries. Many of these countries are in sub-Saharan Africa, which is the region where the unmet need was highest (24%), double the world average [3, 8]. Unmet need for family planning levels more than 20% are considered as high, and less than 10% are regarded as low according to UN world family planning highlight of 2017 [9].

It was estimated that nowadays, more than 142 million married women in developing countries have an unmet need for family planning or want to prevent pregnancy [9]. Because of lack of knowledge, low accessibility of modern contraception, preference of many children, and many other reasons married women and their partners are not using contraception [10].

According to a report from the Ethiopian government, so far, the government of Ethiopia made efforts to reduce the unmet need for family planning to 10% at the end of 2015 by aligning the Health Sector Development Plan IV (2011-2015) with millennium development goals. However, according to the Ethiopian demographic health survey (EDHS) 2016 report, still 22% of married reproductive age women have had an unmet need for family planning. In addition to the high unmet need for family planning, there were also variations among different regions of the country [11–14]. To address this gap in the current Health Sector Transformation Plan and Reproductive health strategy (2016-2020) of Ethiopia, there is an expected target of reducing unmet need for family planning from 22% to 10% among reproductive-age women [11, 15].

To reduce the magnitude of the unmet need for family planning, evidence on magnitude and factors affecting unmet need are crucial. Although there were some studies conducted previously in Ethiopia on the prevalence and associated factors of unmet need for family planning among married reproductive-age women, there may be differences in sociodemographic, cultural, and access to health care factors among the study participants. The current study will help in delineating the individual, community, and health services level factors that can be harnessed to decrease the unmet need of family planning. Therefore, this study was aimed at assessing the associated factors for the unmet need for family planning among currently married reproductive age women in Toke Kutaye District, West Shoa Zone, Oromia region, Ethiopia.

## 2. Method and Materials

### 2.1. Study Design, Period, and Setting

Across sectional study was conducted at Toke Kutaye district. Toke Kutaye is one of the districts in the West Shoa zone of Oromia Regional State, Western Ethiopia, which is located 114 km from Addis Ababa. The district is divided into 23 rural and 4 urban kebeles. According to the report from the District Health office, the total population of the district was estimated to 124,146 in 2019 G.C. According to the District Health office, the sex distribution was 60,832 males and 63,314 females [16]. A community-based quantitative and qualitative cross-sectional study design was conducted in March 2019 among 494 married reproductive age women selected by systematic random sampling from 9 kebeles of Toke Kutaye district. The qualitative study was intended to explore the sociodemographic and cultural factors that affect family planning utilization and unmet need for family planning. The findings were triangulated with the qualitative result.

### 2.2. Source and Study Population

#### 2.2.1. Source Population

All currently married women of childbearing age (15-49 years old) who were residing in Toke Kutaye district.

#### 2.2.2. Study Population

Currently married women of childbearing age were selected by systematic random sampling from 9 kebeles of Toke Kutaye district from whom actual data was collected.

### 2.3. Sample Size Determination

The sample size was calculated using the formula for a single population proportion manually considering the assumptions of 95% confidence level, the margin of error (0.05), *p* = 0.265 (prevalence of unmet need for family planning among married women in a study conducted in Misha district) [17].

The Sample size was determined using a formula *n* =  ((*Zα*/2)^2^ *P* (1 − *P*)/d^2^ .

In calculating the sample size, 10% nonresponse rate, and design effect of 1.5 was taken into consideration, thus, making the total sample size 494.

### 2.4. Sampling Technique

Based on the number of reproductive-age women in each kebele, samples were allocated to each kebele proportionally. Then, the required sample size of 494 was selected proportionally from all selected kebeles by the systematic random sampling technique. The lists of households (HHs) as a sampling frame were obtained from the health extension workers (HEWs), which were registered for trachoma mass drug administration purposes.

For the qualitative study, the purposive sampling technique was used to select study subjects. Four focused group discussions married reproductive age women were participated (6 women in one group) from the same kebeles where the quantitative data were collected.

### 2.5. Study Variables

The dependent variable of this study was the unmet need for family planning which is the sum of unmet need for spacing and unmet need for limiting. The independent variables were socioeconomic variables , reproductive health factors, and family planning factors. In this study, demand for family planning is the number of women with an unmet need plus the number of currently using contraception (representing “met need”). The demand satisfied is referring to the proportion of women using contraception divided by the number of women with demand for family planning. And demand not satisfied is measured by the proportion of women with unmet needs divided by the total contraceptive demand.

### 2.6. Data Collection Tool and Procedures

For the quantitative study, a survey questionnaire was adapted from different literature which was developed for similar purposes by different researchers. The Westoff model 2012 and EDHS report of 2016 (15, 35) were used in developing a tool for the study. Westoff model which is one of the best models to measure the unmet need for family planning is used in measuring the unmet need for family planning in this study [15]. For the quantitative study, data collection instruments were an interview guide. By using a structured questionnaire, married reproductive age women from the selected source population were interviewed by trained data collectors in Afan Oromo. For the qualitative study, a Focus Group Discussion and a semistructured open-ended interview guide for key informants were prepared. Supervision was conducted during data collection. All study participants were told that their responses were kept secret and used for the research purpose only. All Focus Group Discussions and Key Informants were tape-recorded, after seeking consent from the participants. The discussion was conducted in Afan Oromo and translated back to English. Next, completed transcription was compared with handwritten notes to fill inaudible phases or gaps in the tape recorder.

### 2.7. Data Quality Control and Management

The questionnaire was prepared in English and translated into Afan Oromo by a language expert and back to English to ensure its consistency. The survey questionnaire was pretested, and some amendments were made to regulate and ensure its validity from piloting the instrument. Quantitative data were first checked manually for completeness and entered into Epi-Data version 3.1 computer software.

### 2.8. Data Processing and Analysis

The entered data were transferred to statistical packages of social science (SPSS) version 21 computer software programs for further statistical analysis. The frequency was used to check missing values and outliers. In univariate analysis, measures of central tendency (mean) and measures of dispersion (standard deviation) for continuous variables were computed. The odds ratio with a 95% confidence interval was computed by binary logistic regression to assess the presence and degree of association between dependent and independent variables. Variables with *p* < 0.25 with their COR and 95% CI in the bivariate analysis were entered into multivariate analysis to control the confounding effects. Finally, *p* value of ≤ 0.05 and AOR with 95% CI were set as a cut-off point for the significance and strength of the association between dependent and independent variables in multiple logistic regression analysis.

Data adequacy was checked by Hosmer & Lemeshow-Goodness of fit-test (*p* value > 0.05). Multicollinearity was also checked.

For qualitative data, the tape-recorded and notes obtained from Focus Group and Discussion and Key Informants were transcribed carefully by the investigator word by word and arranged with the written notes taken at the time of discussion and interview. The data were grouped based on thematic frameworks (thematic areas). Concepts were extracted from themes and presented in narratives, and data were categorized and analyzed thematically. Through this process, a verbatim quotation was used to illustrate responses on relevant issues and themes. The results were presented in narratives and triangulated with the quantitative information to answer the research objectives question.

### 2.9. Ethical Consideration

Ethical clearance was secured from the Ethical Clearance Committee of College of Medicine and Health Science, Ambo University. An official consent letter was issued from Toke Kutaye's administrative health Office. Verbal informed consent was obtained from each participant. Confidentiality was maintained at all levels of the study.

## 3. Results

### 3.1. Sociodemographic Characteristics of the Study Population

A total of 494 currently married reproductive age women from 7 rural and 2 urban Kebeles of Toke Kutaye district were included in a study with a response rate of 100%. Three hundred twenty-seven (67.2%) respondents were from rural kebeles. The mean age of the respondents was 31.1 (±6.4) years. The mean of respondents' age at first marriage was 19.9 (±2.8) years. The great majority of the women were Oromo 475 (96.2%), and the rest were Amhara 19 (3.8%) by ethnicity (Table 1).

### 3.2. Reproductive Health Characteristics of the Study Population

Among respondents, 461 (93.3%) were ever been pregnant, among these, 451 (98%) ever gave birth. Sixty-one (12.3%) of respondents were currently pregnant at the time of data collection. One hundred sixty-eight (37.3%), 139 (30.9%), and 143 (31.8%) women had 1-2, 3-4, and, ≥5 total living children, respectively. Sixty-one (13.2%) of respondents had a history of child death. Sixty-six participants (14.3%) had a history of abortion, and of these, 57 (83.8%) had an abortion once and the rest two times and above (Table 2).

The qualitative finding augmented that majority of the discussants mentioned that the ideal desire of their family size would be four children. Spacing or limiting family size is important to provide sufficient food, send to school, and purchase clothing, and fulfill other needs for children. Discussants were also explained that due to lack of satisfying unmet need for family planning some mothers were exposed to unplanned birth or large family size. On the contrary, some participants have explained an opinion of the number of family size depends on wealth status. One of the discussants (28 years' woman) shared her experiences on family planning as follows.

“I have four children, I was given birth of two children consecutively within two years, and I was caring them like twins, because of this, I suffered a lot with them. After I have got awareness and started to utilize Family Planning, I gave birth to the third child in three years then I gave birth to the fourth child after spacing of six years, since eight years I have stopped. Four children are enough, adding another child will be a burden for me and also for the child.”

#### 3.2.1. Current Contraceptive Use Pattern of the Study Population

The current survey revealed that 205 (41.5%) of the respondents were using contraceptive methods at the time of data collection. Among current users, 173 (35.0%) and 30 (6.1%) were used for spacing and limiting, respectively. The most commonly used methods were injectable 85 (41.5%), followed by implant 68 (33.2%).

#### 3.2.2. Reasons for Not Using Family Planning Methods

**Figure 1:**
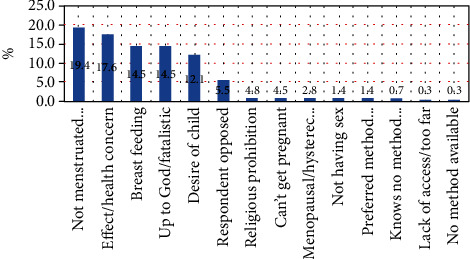
Reasons for not using family planning among nonusers reproductive age married women Toke Kutaye district, West Shoa, Oromia, Ethiopia, March 2019.

The reasons for not using family planning for nonusers were not menstruated 19.4%, religious prohibition and up to God (being fatalistic) 19.3%, fear of side effects/health concerns 17.6%, due to breastfeeding 14.5%, desire of child 12.1%, and others reasons 17.1% (Figure 1).

The qualitative finding was also intended to assess reasons for not using family planning. Accordingly, fear of side effects, poverty (using family planning requires “good nutrition”), desire to have children, religious prohibitions, husband's disapproval, and lack of adequate awareness about family planning were some of the reasons why the discussants were restricted to use family planning methods.

38 years old currently married woman discussants further explained the side effects of family planning from other user's experience as shown in the following extract.

“Last time while I was in health center a man who came with his wife told us his wife was a user of a Family Planning method which inserted in her uterus (IUCD) for five years. After the insertion of IUCD, she could not do her duty properly. She complains of sickness and now she is very ill, now I came with her he said. Using contraception for child spacing is acceptable, but the side effects are a serious problem. When the service of Family Planning was expanding everywhere both females and males were very happy.

But currently because of its side effects and health problems mothers are complaining to discontinue. For me, it is better if she gives birth and suffers with difficulties of child protection rather than by using Family Planning methods and suffers from different health problems.”

There were a few women and men who said that it is up to “God” to determine the number of children, and children are assets.

### 3.3. Unmet Need for Family Planning among the Study Population

**Figure 2:**
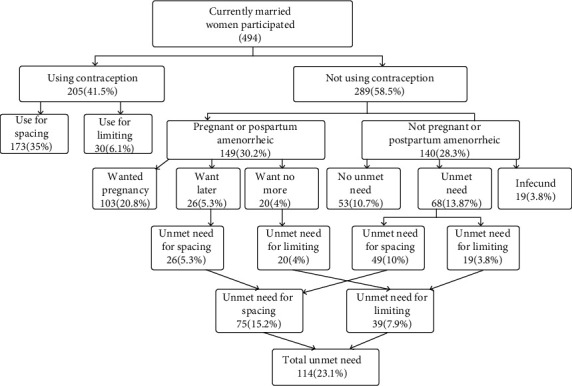
Unmet need for family planning among married reproductive-age women, Toke Kutaye district West Shoa zone, Oromia Region, Ethiopia, March 2019 (Westoff Model, 2012).

The unmet need for modern family planning was found to be 114 (23.1%) [95% CI (19.2, 26.7)], with 75 (15.2%) and 39 (7.9%) were for spacing and limiting, respectively (Figure 2). The percentage of demand satisfied for modern family planning was 64.3%. If all currently married women who have an unmet need for spacing and limiting of family planning were using family planning methods, the family planning prevalence in this study area would increase from 41.5% to 64.6%.

The qualitative study approved the consequences of unmet need family planning like unwanted and mistimed pregnancies. But some community members lack awareness and do not use the family planning services. Most discussants were very aware of the need for avoiding unwanted and mistimed pregnancies through family planning services.

Accordingly, discussants of the Focus Group Discussion explained that mistimed and unwanted pregnancies were happened due to not to use and improper use of family planning methods, contraception failure, poor counseling of health workers, the delay from revisiting or repeat, discontinue of family planning, and irregularities of menstruation occurrences. These have exposed many mothers to an unplanned pregnancy and a cause even for abortion and health problems.

The Key Informant participants said that the utilization of contraceptive methods in their kebeles shows progress from time to time. Because they have provided health education, the awareness of the community is increasing. But, there is a challenge that some women still have not utilized it. This exposes mothers to unwanted and mistimed pregnancies, which have occurred because of unmet need family planning among married reproductive-age women. The participants had also reported that some mothers were not willing to use family planning timely after birth due to misunderstanding of waiting to use family planning until their menstruation begins. In such conditions, unwanted pregnancy happened occasionally. To this regard, 11 years experienced health extension service worker witnessed:

“I knew a woman who gave birth of another baby within 11-month interval; while she was waiting for menstruation to start Family Planning she exposed to unwanted pregnancy.”

### 3.4. Family Planning Factors

Concerning the knowledge level of the respondents, 310 (62.8%) had good knowledge about family planning methods and their sources. The majority of the respondents 429 (87%) knew at least one method of contraceptive. From those who knew some methods of family planning, 291 (76%) knew injectable contraceptive followed by 263 (68.5%) implants, 237 (48.0%) oral contraceptive pills, and IUCD 217 (56.5%).

The qualitative finding was also in line with the quantitative study concerning knowledge of discussants about family planning methods. The majority of married women discussants revealed that utilizing contraception methods is important for satisfying unmet need for family planning. As they revealed in the Focus Group Discussion, most of them used these methods. For instance, they had mentioned the name of some contraceptives like injection (Depo Provera), pills, uterus/loop (IUCD), the closing of the uterus (female sterilization), and implants.

Even though the majority of discussants have good knowledge status of family planning methods and sources, still some of them had poor knowledge status on how to satisfy their need for contraceptives.

24 years old currently married woman discussant said: “I have no education, I haven't any information concerning advantage and disadvantages of Family Planning; no awareness is given for both men and women in my village, so I am not currently utilizing it.”

Three hundred eighty-three (77.5%) reported they knew that the sources of modern family planning were health facilities in the district. Of these, 307 (80.2%), 298 (77.8%), 219 (57.2%), 169 (44.1%), and 51 (13.3%) of the respondents mentioned health posts, health centers, hospitals, private clinics, and drug vendor, respectively, as the source of modern family planning. Three hundred forty (69.0%) respondents got information about family planning from health workers followed by radio and/or TV 306 (62.0%) and friends 159 (32.2%).

The qualitative study assured that most of the participants reflected as they had knowledge of family planning methods and mentioned their sources of family planning information were health extension workers, other health workers, and their friends. They also stated as sources of family planning methods were health posts, health centers, and private clinics. However, both FGD and KI participants did not report the shortage of family planning supply in their area. They have also explained the possibility to be referred to the nearby health center for long-acting.

Of the total respondents, 352 (71.3%) of mothers discussed contraceptive methods with health care providers, and 269 (54.5%) visited healthcare facilities within the last 12 months before this study. Only 202 (40.9%) participants were discussed with their partner about family planning six months before the survey. Three hundred thirty-two (67%) of their partners have supported the use of family planning (Table 3).

The qualitative study also identified that family planning utilization was less in those mothers who were not made discussion with Health Workers, not frequently visiting health facilities, and their husband not support her partner to use family planning. Women discussants have confirmed as healthcare providers frequently providing awareness about family planning advantage and related issues to them. Thus, most mothers were practicing their advice. But, those mothers who resist practicing health extension advice, have no contact with them, and cannot visit health facilities are not still utilizing family planning.

Regarding this, one of the Focus Group Discussion participants (35 years old married women) reflected as follows.

“I have no time to participate in health extension meetings and also not have visited health facility due to workload. I am not currently utilizer of Family Planning due to my problem.”

### 3.5. Associated Factors of Unmet Need for Family Planning

Candidate variables with *p* value < 0.25 at 95% CI were entered into a multivariate logistic regression to control for the confounding variables and establish statistical significance using the forward stepwise likelihood ratio method (Table 4). Variables that had *p* ≤ 0.05 at 95% CI, were considered as significantly associated. Accordingly, variables like mother educational status, number of living children, and discussion with a healthcare provider on family planning and husband support of family planning were found significantly associated with unmet need for family planning.

Married women who have no formal education were about 3.6 times [AOR = 3.64, 95% CI: 1.43-9.25] found to be more likely to have an unmet need for modern family planning than married women who have secondary and above educational status. Those women who have five and more living children were about 2.6 times [AOR = 2.63, 95% CI: 1.37-5.05] more likely to have an unmet need for family planning than currently married women who have 1-2 living children. Married women who had discussed family planning issues with healthcare providers within the last year were 80% less likely to have an unmet need for family planning than their counterparts [AOR = 0.20, 95% CI: 0.13-0.37]. The husband who disapproves of his wife using family planning was about 3.7 times more likely to have an unmet need for family planning compared to the husband who approves his partner to use contraception [AOR = 3.68, 95% CI: 2.20-6.16] (Table 4). In multivariable logistic regression model residence of women, number of pregnancies, number of son children, having TV and/or radio, and husband knew or not knew using family planning were not significantly associated with unmet need for family planning in this study.

## 4. Discussion

According to the revised West off, the model of 2012, the total unmet need for family planning in this study was 23.1% with 15.2% for spacing and 7.9% for limiting. These findings were still higher than similar studies conducted in Iran Khuzestan province 13% [18], Botswana 9.6% [19], Minia governance of Egypt 12.7% [20], and Dangila town of Amhara 17.4% [21]. This difference might be due to the differences in socioeconomic, awareness status, and attitudes of mothers on contraceptives. On the other hand, the proportion of unmet need for family planning in this study was lower than similar studies conducted in Eastern Sudan 44.8% 26, Burkina Faso 40.7% [22], Butajira 52.4% [23], Arbaminch Zuria district 34.4% [24], Oromia Region 41.3% [25], and EDHS report of Oromia Region 29% [14]. This variation may be due to the expansion of health facilities and improved access to health services including family planning services currently in Ethiopia, expansion of health extension programs, the variation of the year of studies, and effort by governmental and nongovernmental organizations. The finding of this study was similar to studies conducted in Misha district 26.5% [17] and Enemay district of Amhara 25.6% [26]. This similarity might be due to better maternal education and discussion with a healthcare provider in all study settings as well as the number of living children with Misha district have a significant association in multivariable analysis.

The educational status of mothers is one of the socioeconomic variables significantly associated with the unmet need for family planning in this study. Married women who have no formal education were 3.6 times more likely to have an unmet need for family planning than women who have secondary and above education. This result is consistent with studies which were done in Butajira, Arbaminch, and Hawasa of Ethiopia and Eastern Sudan, which revealed that those mothers who have no formal education had increased unmet need for family planning than those who have more educational status. Besides, unmet needs progressively declined with increasing levels of education [23, 24, 27, 28]. The possible explanation for this could be those mothers who have no formal education could not read and understood different messages of family planning in written-words and social media to improve their knowledge, have less access to health facilities, and exposure to sources of information. This finding was also supported by Focus Group Discussion conducted with married women which explained that mothers who have no formal education have low access to information sources of family planning.

The number of living children is one of the reproductive factors which were significantly associated with the unmet need for family planning in this study. Currently, married women who have five and above living children were found to be about 2.6 times more likely to have an unmet need for family planning than women who have two and fewer children. This result is in agreement with similar studies done in Ethiopia [2, 25] Uganda [29] in low and middle-income countries [30]. All these studies reported that the number of living children is strongly and significantly associated with the unmet need for family planning, indicating that as the number of living children increases, the unmet need for family planning also increases. The possible reasons for having many living children are low awareness of mothers, having many children for sharing labor and household responsibilities, and religious prohibition. The qualitative study findings also assured that religious prohibition, traditional beliefs, poor knowledge, and the need for child labor were identified as reasons for wanting many children or a large family.

The other predictor variable which was significantly associated with the unmet need for family planning in multivariable logistic regression was discussion or counseling given by health workers and/or health extension workers in the last year. Currently, married women of reproductive age who have made discussion or counseled by healthcare provider were 80% less likely to have an unmet need for family planning than women who had not made a discussion with a healthcare provider on family planning.

This result is similar to a study which was done in Dangila town which indicated that women who were not counseled about contraception were more likely to have an unmet need for family planning when compared to women who were counseled [21]. Other similar studies were done in Shire Enda Sillaise [31] and Misha district [17] also revealed that the unmet need for family planning increases in those women who made no discussion with health workers. It might be because women who have made discussions with healthcare providers were counseled properly about family planning advantages, methods choices, and its source. The qualitative study explained that mothers who did not visit health facilities frequently and less contact with health care providers did not utilize family planning.

Disapproval of husband on family planning was significantly associated with unmet need for family planning. Those married women whose husbands disapprove of the utilization of family planning were about 3.7 times more likely to have an unmet need for family planning than married women whose husbands approve of family planning utilization. This factor has been proven by many studies to have high significant associations with the unmet need for the family planning region of Dangila [19–21]. Women whose partners had a nonsupportive attitude for family planning use were more likely to have an unmet need for family planning. This may be due to the decision-making power of the husband is higher than his partner in all aspects of his family's life including family planning utilization in our society and also may be due to the desire of many children, fear of side effects, and infertility. This result is also supported by Focus Group Discussion conducted with married men. As it was stated by a 26-year old man who was among the target participant asserted even if he knew about family planning and also his wife has the interest to use the opposed her not to use due to wanting of additional children and rumor of side effects. This is also supported by the key informant participants as they were stated that some women have no right to decide to use family planning.

## 5. Limitations of the Study

Since the design is cross-sectional, temporal relations could not be assessed. The study may be affected by recall bias because it was retrospective and may be susceptible to social desirability bias.

## 6. Conclusion and Recommendation

Despite many efforts were made to reduce the unmet need for family planning by expanding access to sources of family planning, a significant proportion of currently married women in the district still has an unmet need for both spacing and limiting of births. The finding of this study revealed that the prevalence of unmet need for family planning was to be 23.1% which was similar to the findings of national and less developing (sub-Saharan African) countries. It was very far from the national target to reduce the unmet need for family planning to 10% by the end of 2020. It is concluded that maternal education, number of living children, husband disapproval of family planning, and making discussion with healthcare provider were significantly associated with unmet need for family planning.

Based on the findings, the following recommendations were given. The health sector and partners should encourage and support health facilities to work in integration on improving utilization of family planning service to reduce unmet need for family planning. The health sector, Education sector, and respective NGOs' should strengthen mother's education especially in promoting basic adult education. District health office and women affair should strengthen their collaboration on empowering women decision-making power on family planning. Family planning providers should encourage men's involvement during counseling or discussion about family planning to enhance joint decision of family planning utilization to reduce unmet need for family planning.

## Figures and Tables

**Table 1:** Sociodemographic characteristics of married reproductive-age women, Toke Kutaye district, West Shoa, Oromia, Ethiopia, March 2019 (*n* = 494).

Characteristics	Category	Frequency	Percent
Educational status of the mother	No formal education	233	47.2
	Primary education	171	34.6
	Secondary and above	90	19.6

Partner educational status	No formal education	112	22.7
	Primary education	226	45.7
	Secondary and above	156	31.6

Occupation of mothers	Housewife	375	75.9
	Merchant	35	7.1
	Daily laborer	29	5.9
	Employed	30	6.1
	Others^∗^	25	5.1

Average monthly income	<1500	350	70.9
	1501-3000	83	16.8
	>3000	61	12.3

Current mother age	18-24	67	13.6
	25-34	253	51.2
	≥35	174	35.2

Owning of media access	None	210	42.5
	Radio only	119	24.1
	TV only	129	26.1
	Both radio and TV	36	7.3

^∗^Others indicate jobless, local drink sellers, and students.

**Table 2:** Reproductive health characteristics of married reproductive-age women, Toke Kutaye district, West Shoa, Oromia, Ethiopia, March 2019; (supplementary table).

Characteristics	Category	Frequency	Percent
Age at 1st pregnancy (*n* = 461)	≤17	59	12.8
	18-20	179	38.8
	21-24	178	38.6
	≥25	45	9.8

Number of pregnancy (*n* = 461)	1-2	164	35.5
	3-4	131	28.4
	≥5	166	36.1

Age at 1st birth (*n* = 451)	≤17	43	9.5
	18-20	158	35.1
	21-24	199	44.1
	≥25	51	11.3

Number of sons (*n* = 450)	0	77	17.1
	1-2	252	56
	≥3	121	26.9

Less than 5 years' child (*n* = 494)	Yes	362	73.3
	No	132	26.7

Number of fewer than 5 years' children (*n* = 362)	1	260	71.8
	≥2	102	28.2

**Table 3:** Family planning factors among currently married reproductive age women in Toke Kutaye District, West Shoa Zone, Oromia, Ethiopia, March 2019; (supplementary table).

Variables	Category	Frequency	Percent
Approve or disapprove of couples (*n* = 494)	Approve	367	74.3
	Disapprove	127	25.7
Reasons for disapproval (*n* = 127)^∗^			
Religion prohibition	Yes	52	41
	No	75	59
Fear of side effect	Yes	93	72
	No	34	28
Medical problem	Yes	60	47.2
	No	67	52.8
The desire for more children	Yes	27	21.3
	No	100	78.7
Husband know family planning (*n* = 494)	Yes he does know	409	83
	No, he does not know	51	10
	I am not sure	34	9
Ever used any family planning methods for not currently using (discontinue) (*n* = 289)^∗^	Yes	171	59
	No	118	41
Want to have another child (*n* = 171)	Yes	49	17
	No	122	83
Fear of side effects (*n* = 171)	Yes	91	31.5
	No	80	68.5
Health/medical problem (*n* = 171)	Yes	45	26
	No	127	74
The need for secrecy (*n* = 171)	Yes	2	1.2
	No	169	98.8
Poverty (*n* = 171)	Yes	5	3
	No	166	93
Contraception failure (*n* = 171)	Yes	4	2.3
	No	167	97.7

^∗^Multiple responses.

**Table 4:** Multivariate logistic analysis of independent variables associated with unmet need for family planning in Toke Kutaye district, West Shoa zone, Oromia, Ethiopia, March 2019.

Variables	Category	COR (95% CI)	AOR (95% CI)	*p* value
Mothers education status	No formal education	6.08 [2.69-13.78]	3.64 [1.43-9.25]	0.007
	Primary education	2.32 [0.97-5.55]	1.53 [0.58-4.02]	0.391
	Secondary and more	Ref.		

Number of living children	1-2	Ref.		
	3-4	1.54 [0.87-2.73]	1.31 [0.68-2.54]	0.417
	≥5	3.41 [2.00-5.82]	2.63 [1.37-5.05]	0.004

Discussion with healthcare provider on family planning in last one year	Yes	0.20[0.12-0.29]	0.20[0.13-0.37]	<.001
	No	Ref.		

Husband support on family planning	Disapprove	5.82 [3.71-9.13]	3.68 [2.20-6.16]	<.001
	Approve	Ref.		

Ref.: Reference.

## Data Availability

The data that support the findings of this study has a sort of identifier of individual participants and researchers reserved to send it.
